# A physiological approach to understand the role of respiratory effort in the progression of lung injury in SARS-CoV-2 infection

**DOI:** 10.1186/s13054-020-03197-7

**Published:** 2020-08-10

**Authors:** Pablo Cruces, Jaime Retamal, Daniel E. Hurtado, Benjamín Erranz, Pablo Iturrieta, Carlos González, Franco Díaz

**Affiliations:** 1 Escuela de Medicina Veterinaria, Facultad de Ciencias de la Vida, Universidad Andres Bello, Santiago, Chile; 2 Unidad de Paciente Crítico Pediátrico, Hospital El Carmen de Maipú, Santiago, Chile; 3 Departamento de Medicina Intensiva, Pontificia Universidad Católica de Chile, Santiago, Chile; 4 Instituto de Ingeniería Biológica y Médica, Pontificia Universidad Católica de Chile, Santiago, Chile; 5 Department of Structural and Geotechnical Engineering, School of Engineering Pontificia Universidad Católica de Chile, Santiago, Chile; 6 Institute for Biological and Medical Engineering, Schools of Engineering, Medicine and Biological Sciences, Pontificia Universidad Católica de Chile, Santiago, Chile; 7 Millennium Nucleus for Cardiovascular Magnetic Resonance, Santiago, Chile; 8 Centro de Medicina Regenerativa, Facultad de Medicina, Universidad del Desarrollo, Santiago, Chile; 9 Unidad de Paciente Crítico Pediátrico, Hospital Clínico La Florida Dra. Eloísa Díaz Insunza, Santiago, Chile; 10 Instituto de Ciencias e Innovacion en Medicina (ICIM), Universidad del Desarrollo, Santiago, Chile

**Keywords:** Mechanical ventilation, SARS-CoV2, COVID-19, P-SILI, Lung strain, Work of breathing

## Abstract

Deterioration of lung function during the first week of COVID-19 has been observed when patients remain with insufficient respiratory support. Patient self-inflicted lung injury (P-SILI) is theorized as the responsible, but there is not robust experimental and clinical data to support it. Given the limited understanding of P-SILI, we describe the physiological basis of P-SILI and we show experimental data to comprehend the role of regional strain and heterogeneity in lung injury due to increased work of breathing.

In addition, we discuss the current approach to respiratory support for COVID-19 under this point of view.

## Background

Severe acute respiratory syndrome coronavirus 2 (SARS-CoV2) pandemic has pushed health systems’ response to its maximum capacity. In many countries, the surge of cases has exceeded the facilities, technological, and human resources availability at all levels of care. Intensive care units have been overcrowded due to swarming of severe cases in a few weeks, where acute respiratory failure (ARF) and acute respiratory distress syndrome (ARDS) are the main cause of admission. Protective low-tidal volume (Vt) mechanical ventilation (MV), including delivering a physiologic low Vt adjusted by ideal body weight, is currently the standard of care for patients requiring invasive respiratory support, like moderate and severe ARDS. The surge of patients presenting with SARS-CoV2 has led to an unprecedented demand of mechanical ventilators, surprising the whole world with a shortage of equipment unthinkable just 6 months ago. Due to high demand of invasive MV in many hospitals, mechanical ventilators have become a scarce or non-existent resource, and other respiratory support strategies have been used, including high flow nasal cannula (HFNC), non-invasive ventilation (NIV), and other alternative devices. Specific indications for their use are not well defined, consensus guidelines are controversial and frequently they are not followed in clinical practice. The risk of healthcare professional’s infection due to aerosolization was suggested as a strong contraindication for HFNC and NIV at the beginning of pandemic, contributing in some degree to the shortage of invasive mechanical ventilators. As pandemic reached peak of cases, use of non-invasive devices became widespread. Cohort studies form China, Italy and North America [1–3], showed NIV use between 10 and 30% of patients, but when considering single center and small case series it ranges from 0 to 69%. Although non-invasive respiratory support may prevent invasive MV, failure of this approach may lead to morbidity and mortality [4–12]. Some patients will remain dyspneic, breathing spontaneously, with or without respiratory support. Currently, indirect information suggests that vigorous and dysregulated respiratory effort may be a promoter of lung injury, a phenomenon known as “patient self-induced lung injury” (P-SILI) [13–15].

## Biomechanical framework for amplification of lung damage: stress and strain

The lung can be described as a pre-stressed network of viscoelastic tissue elements deformed by surface tension and the action of respiratory musculature. This characteristic allows deformation in a time-dependent manner upon applied pressure and return to its initial configuration once the pressure is relieved [16]. Breathing produces a phenomenon of continuous cyclic strain deformation throughout life, where the applied pressure is inspiratory pressure. In biomechanical terms, deformation in the lung is measured in terms of *strain*, defined as the relative change in volume normalized by a reference volume. This biomechanical property can be defined for the whole lung (global strain) as the ratio between the Vt and a reference volume, usually the volume of air at the end of passive expiration, and the functional residual capacity (FRC). Correspondingly, the force acting on a surface unit, producing its deformation, is the *stress*. The transpulmonary pressure corresponds to the stress in the lung. Strain and stress in the lung tissue are closely related to each other through a constitutive relation (stress = tissue elastance*strain). Both are considered to play an important role in the onset and development of ventilator induced lung injury (VILI). High values (non-physiological) of strain, measured as pulmonary tissue deformation relative to volume change, are known to be harmful to the lung and to increase mortality in ARDS patients under MV [17]. Indeed, improved clinical outcomes observed in ARDS patients due to lower Vt corresponds to a reduction of the lung deformation because of MV [18]. These compelling and well-established findings have directed the attention of several groups to understand the regional mechanisms of deformation in mechanically ventilated patients. Understanding the global strain in the lung has allowed the identification of thresholds of safer Vt to prevent VILI, currently present in guidelines and consensuses [17].

## Mechanotransduction: coupling biochemical response and applied energy

In injured lungs, there is a wide spectrum of tissue aeration, producing inhomogeneity of ventilation. Lung inhomogeneity has been recently proposed as a VILI promoter in ARDS patients, given the fact that lung injury can occur despite the use of recommended Vt and pressures, parameters that are considered to be safe in the ventilation of healthy lungs. The concept of stress raisers may explain these findings. The term stress raisers refer to those additional regional factors capable of intensifying the damage. Stress raisers produce amplification of the stress applied in certain localized regions of the lung, like the areas of high inhomogeneity of ventilation [19–22]. The deleterious effects of high regional strain in the lung was confirmed recently in a swine model of injurious MV, where lung zones of increased regional strain had a spatial correlation with areas of tissue inflammation [23]. This study highlights the relevance of a better understanding of the spatio-temporal progression of regional strain, supporting that strain is a relevant and prominent determinant of VILI [23–25]. The heterogeneous distribution of opening pressures throughout the lung results in an overstretch of the aerated lung zones (“baby lung”) and also in collapsed (poorly aerated) regions due to repetitive cycles of recruitment–derecruitment. The generation of injurious mechanical forces is inevitable when invasive MV is applied, due to the heterogeneous nature of ARDS and the inflation/deflation dynamics of the lungs. There is a coupling between the applied mechanical stimuli and the biochemical response of lung cells, a biological process called mechanotransduction [26, 27]. Mechanotransduction can be a pathway of lung injury when the mechanical stimuli are excessive, triggering an inflammatory response in the lung. Amplification of lung damage, i.e., VILI, depends on the level of energy dissipated by the lung parenchyma and its deformation. The lung does not discriminate the origin of these forces that can be generated by MV or by the respiratory muscles. In this way, biomechanical mechanisms that cause P-SILI can occur with or without MV.

## P-SILI during mechanical ventilation

There is strong evidence that spontaneous ventilation during MV has a role in progression of lung injury [28]. Although spontaneous breathing has proved beneficial in the treatment of mild ARDS patients, opposite effects occurred when lung injury was severe. Spontaneous breathing amplified the damage in severe lung injury, increasing transpulmonary pressures, atelectasis, cyclic collapse, and histological signs of damage [28–32]. The paradox of spontaneous breathing and lung damage can be explained by the solid-like biomechanical behavior of injured lungs. Some of the mechanisms described for lung injury from spontaneous effort are increased lung stress/strain, increased lung perfusion, and patient ventilator asynchrony. The generation of vigorous diaphragm contractions induces high negative pleural pressures that will be dissipated along the visceral pleura surface in a homogeneous shape (fluid behavior) in case of healthy lungs, but this dissipation is uneven in case of ARDS lungs and stress is concentrated in the interphase of collapsed and ventilated lung (solid behavior). This increment in local lung stress has been associated with higher lung inflammation in the dependent lung regions in experimental models. In addition, increment of venous return and oscillations in pulmonary blood flow could favor lung edema production, and finally patient-ventilator dyssyncronies as reverse triggering are associated with increments of Vt that may induce VILI [30, 33, 34].

## P-SILI without positive pressure ventilation

There has been a particular emphasis on interventions to prevent MV in recent years, such as HFNC and NIV, maintaining spontaneous ventilation and avoiding VILI [35–39]. Experimental studies and indirect clinical information have given a counter point to this approach, suggesting that spontaneous unregulated ventilatory effort for extended periods of time can also induce progression of the lung damage [13, 14]. In spite of these facts, it may be counterintuitive the current recommendation to avoid or deliberately delay the start of the MV.

Currently, the knowledge of P-SILI in extubated patients is limited. P-SILI occurs in healthy lungs without MV, in some conditions, like an intense increase in minute ventilation (⩒E). Stress failure of blood-gas barrier after forced training in racehorses was described by West et al. in 1993 [40]. Similar findings have been described in elite athletes after prolonged high intensity exercise (i.e., triathletes, marathon runners, and swimmers), which in fact can led to pulmonary edema, in absence of cardiac alterations. After intense exercise, bronchoscopic samples have found higher concentration of red blood cells, total proteins, albumin, and inflammatory cells (neutrophils), mimicking the findings in other mammals [41–44]. These alterations can be correlated to the ones described by Mascheroni et al. in an experimental ovine study [45]. The authors observed a serious deterioration in pulmonary function after 3.5–13 h of pharmacologically induced hyperventilation in spontaneously breathing animals without lung disease. These alterations were prevented by MV and sedoparalysis. This study confirms that vigorous spontaneous ventilation can affect the lung and controlled MV can prevent or attenuate the damage of the lung in this setting [45]. The alterations in lung function in this experiment were inversely proportional to the exposure time to hyperventilation. As authors point out, they could not discriminate if only the “mechanical stress” was responsible for these observations. Off note is that during the observation period, animals were intubated (infraglotic artificial airway) and without positive pressure ventilation. This experimental design may have contributed to the deterioration of lung function by promoting lung atelectasis. For example, Hedenstierna et al. described that perioperative atelectasis collapse can easily reach 50% of the total lung tissue after a few minutes even in uneventful anesthesia [46]. Atelectasis could contribute to P-SILI by two main mechanisms: reduction of FRC and subsequent increment in dynamic strain during tidal ventilation and generation of heterogeneous lung tissue [17, 19, 47, 48].

**Fig. 1:**
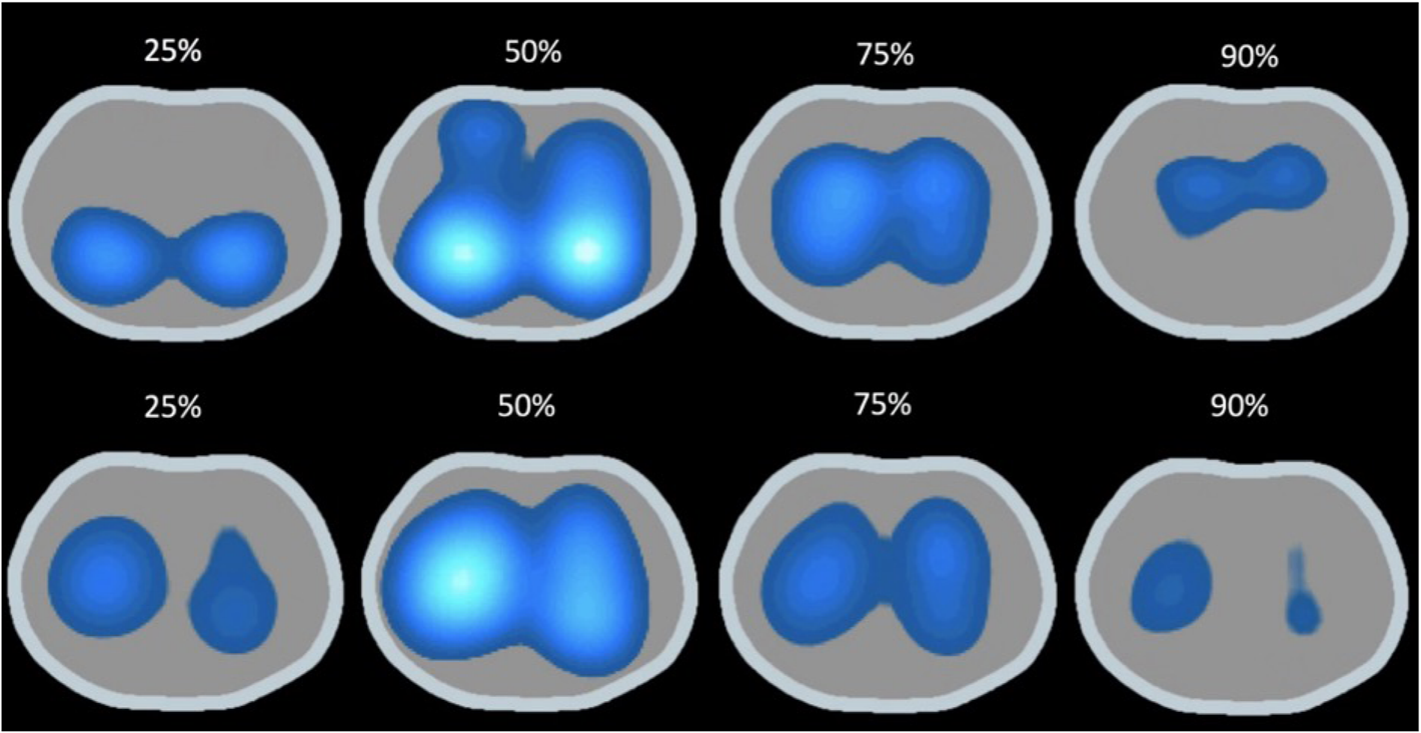
Electrical impedance tomography maps of ventilation in one ARDS patient before (superior line) and after intubation using volume-controlled ventilation (VCV) mode (inferior line). Images correspond to one complete respiratory cycle, and it was divided since beginning of inspiration to end-expiration. Observe differences in the inflation pattern. At beginning of inspiration ventilation starts in the most dependent lung regions in spontaneous breathing (SB) and more central regions in VCV. At the end of inspiration ventilation is predominantly dorsal in SB and predominantly central in VCV. The beginning of expiration presents a displacement of center of ventilation directed to ventral in SB and maintaining central in VCV. At the end of expiration, it is observed that ventilation ended in the ventral part of lungs in case of SB, and in the intermediate lung region of VCV. Modified from: Bachmann MC, Basoalto R, Soto, et al. Intensive Care Med Exp. 2018;6(Suppl 2):0274

Recently, we developed a 4D tomographic study that employs image-based biomechanical analysis [49] to unveil the volumetric distribution of regional deformation of the whole lung in subjects without MV. In healthy sedated rats under (unassisted) spontaneously breathing, we observed volumetric regional strain and strain heterogeneity, quantifying the magnitude of these deformation indices and its progression in time [50]. Given the fact that regional strain and heterogeneity are present during a normal respiratory cycle without harming the lung leads to the question: why P-SILI does not develop in normal lungs deformed by physiologic Vt? The answer probably is related to many factors, as the amount transpulmonary pressure generated, alveolar–capillary barrier indemnity and the magnitude and topographic distribution of dissipated energy on the lungs. A possible explanation might be that the susceptibility to P-SILI depends on the size of the FRC, prior to injury induced by high global strain. Loss of normally aerated lung volume has two main effects: less lung available for tidal deformation and increased force of diaphragmatic contraction. For a same Vt, a lung with lower FRC is inherently more susceptible to global regional strain. Reduced lung volume has important effects on diaphragm position and function. Cephalad displacement results in a greater curvature of the diaphragm and an increase in the size of the zone of apposition. Further, diaphragmatic fibers are lengthened, augmenting its capability of generate force during the contraction. If respiratory neuromuscular function is intact, then increased drive translates into stronger diaphragm contraction and larger “swings” of negative pressure. This has been demonstrated in laboratory studies, in which spontaneous effort was greater in more severe lung injury. Stronger spontaneous effort is linearly related to larger degrees of Pendelluft, as well as greater tidal recruitment and regional strain (Fig. 1).

**Fig. 2:**
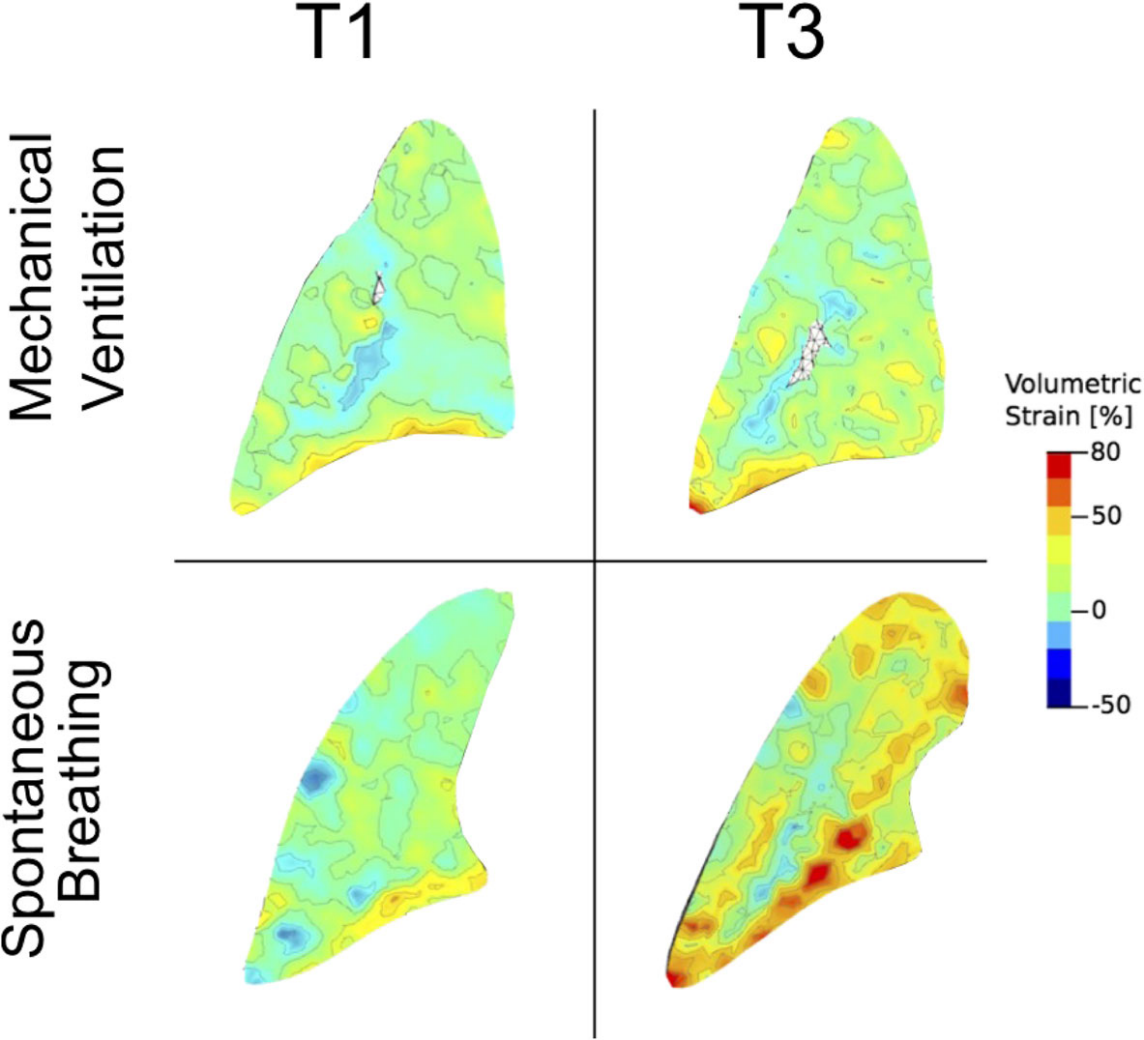
Regional volumetric strain maps in a 3-h murine model of patient self-inflicted lung injury randomized to two groups: Group I: subjects with induced lung injury on low tidal volume mechanical ventilation at the beginning of the experiment (T1) and at the end of the experiment (T3) (upper left and right panels). Group II: subjects with induced lung injury on spontaneous breathing (no mechanical ventilation) at the beginning of the experiment (T1) and at the end of the experiment (T3) (lower left and right panels). Progression of regional strain and heterogeneity in time is observed in spontaneous breathing, which reaches volumetric strain levels of up to 80%. Regional strain distribution remains more uniform and homogeneous in low tidal volume mechanical ventilation

In a follow-up experimental study, we compared animals with acute lung injury under controlled MV and spontaneously breathing without MV. Lung injury was induced by lung lavage in rats, followed by 3 h of spontaneous breathing or low Vt-MV. Micro-CT images were acquired at the beginning and at the end of the observation period, and 4D regional strain maps were constructed. We found a marked tomographic progression of the nonaerated-tissue compartment, and a reduction of the normal-tissue compartment, in accordance to de-recruitment phenomenon. Additionally, we found a significant progression of regional volumetric strain and heterogeneity after spontaneous breathing. In contrast, low Vt-MV had limited progression of the regional strain and heterogeneity at the end of the study (Fig. 2) [51]. Lung heterogeneity has been associated with ARDS severity and mortality [19]. Peri atelectatic alveoli, as Mead et al. described in a theoretical model of alveolar interdependence, can concentrate tension until 4-times in comparison with the global tension applied to the system [52]. Some years ago, our group showed that the peri-atelectatic region in a rat-model of injurious MV presented more inflammation and alveolar disruption than the rest of lung [53]. If we project the alveolar interdependence to heterogeneous lung with multiple collapsed regions, we can explain this as a trigger of inflammation during spontaneous ventilation.

**Fig. 3:**
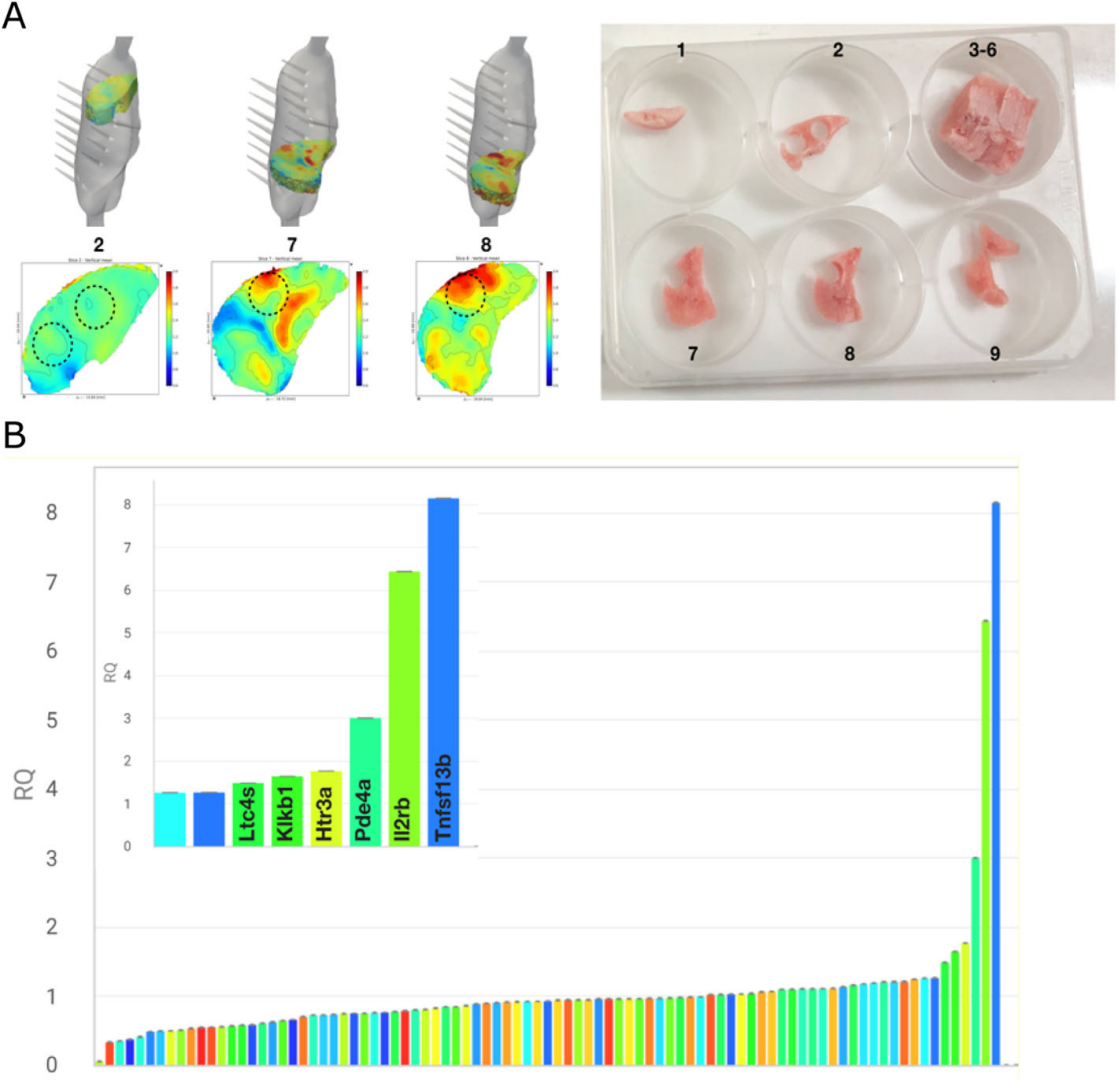
Variation of gene expression in high strain and low strain regions of the lung in a murine model of patient self-inflicted lung injury. **a** Representative images of in vivo/ex vivo fit between tomographic maps of regional strain and 3D digitized frozen lungs. Red areas represent high strain regions, while the green/blue areas represent low strain regions in spontaneous breathing. Low and high strain regions from the same frozen lung were cut, homogenized, and the RNA purified. **b** Gene expression variation of inflammation/pathological mechanotransduction between regions of high and low regional strain. The genes that increased their expression in regions of high deformation were TNF Superfamily Member 13b (Tnfsf13b, > 8 times), Interleukin-2 receptor subunit beta (Il2rb, > 6 times), Phosphodiesterase 4A (Pde4A, ~ 3 times), 5-hydroxytryptamine receptor 3A (Htr3a), Plasma kallikrein (Klkb1), and Leukotriene C4 Synthase (Ltc4s). These genes are involved in apoptosis, IL-2 signaling, G-protein signaling, activation of ligand-activated ion channels, coagulation, and inflammation, respectively

**Fig. 4:**
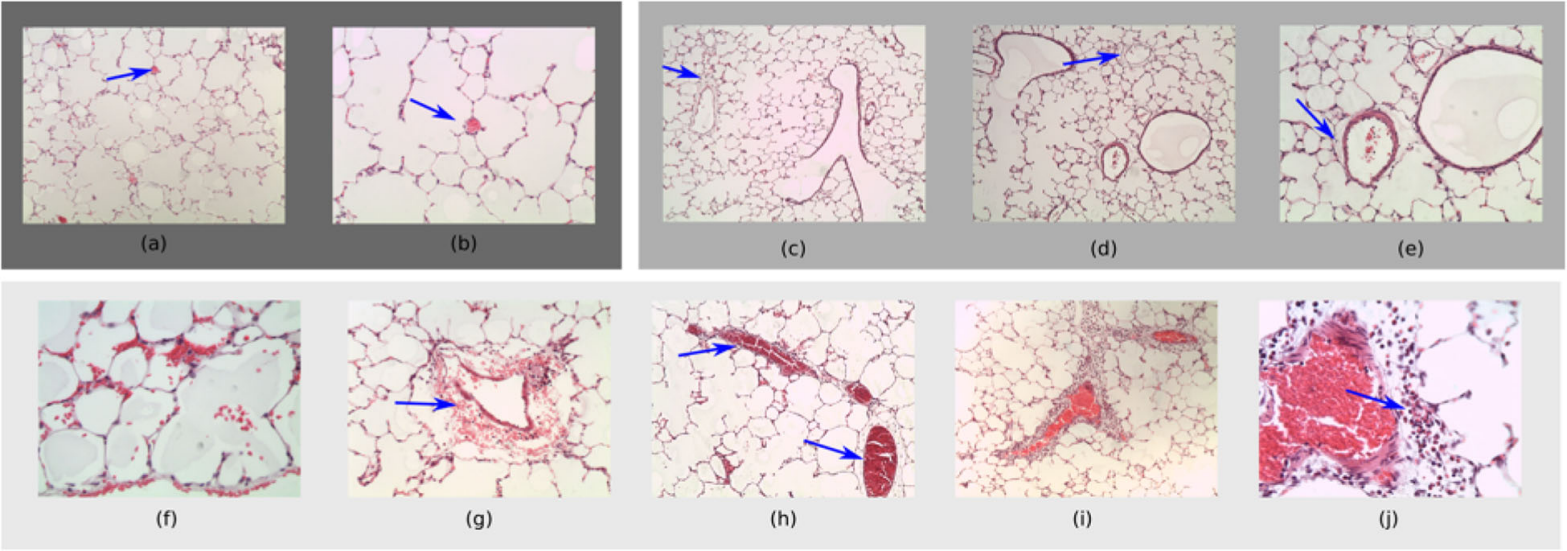
Representative images of lung histology of a 3-h murine experimental study where subjects were randomized to three groups: Group I: subjects with normal (uninjured lungs) on spontaneous breathing (no mechanical ventilation) (**a**, **b**). Group II: subjects with induced lung injury on low Vt mechanical ventilation (**c**, **d**). Group III: subjects with induced lung injury on spontaneous breathing (no mechanical ventilation) (**f**–**j**). In the first image set, no edema or perivascular infiltration is appreciated at ×100 (**a**) and ×200 (**b**). In the second image set, minimal amount of perivascular fluid is occasionally observed at ×100 (c–e). In the third image set, we observed alveolar wall disruption and hemorrhage at ×400 (**f**), perivascular edema and hemorrhage at ×200 (**g**), intense hyperemia in lung parenchyma vascular bed with signs of initial perivascular edema and leucocyte infiltration at ×200 (**h**), intense hyperemia and perivascular accumulation of leucocytes at ×100 (**i**), and perivascular accumulation of polymorphonuclear cell leucocytes and lymphoid cells at ×400 (**j**)

Our group is currently working on topographic correlation of areas of strain and inflammation in the P-SILI model. We measured gene expression pathways on lung tissue homogenate and lung histology. Preliminary results are supportive of our hypothesis. Regions-of-interest (ROI) with high regional strain had increased expression of genes involved in apoptosis, IL-2 signaling, G-protein signaling, activation of ligand-activated ion channels, coagulation, and inflammation, among others, compared to ROIs with low regional strain (TaqMan™ Array Rat Inflammation 96-well plates, Cat. No. 4414081, Thermo Fisher Scientific, USA) (Fig. 3). A similar gene expression was identified in areas of high stretch in mechanically ventilated rats in a high global lung strain model [24]. Off note is that in our P-SILI model, animals under spontaneous breathing had higher degree of histopathological damage compared to low Vt-MV, specifically alveolar wall disruption and hemorrhage, hyperemia, and leucocyte infiltration (Fig. 4). Interestingly, although biomechanical phenomena and gene expression are regional, lung damage was diffuse. A possible explanation for this is that many of the biomarkers mentioned are water soluble and easily diffusible in plasma and respiratory secretions, so they can be secreted locally, but their consequences are more diffuse, and even at distance.

## Rationale of non-invasive support in ARF due to SARS-CoV2: hypothetical fear vs common practice

Gattinoni et al. recently described two phenotypes in patients with SARS-CoV2, “non-ARDS” type 1 (or type L), and ARDS, type 2 (or type H) [12, 54]. Type 1 refers to initial COVID-19 pneumonia, characterized by low elastance, low V/Q ratio, low lung weight, and low recruitability. On the contrary, type 2 fulfills classic criteria of ARDS. In a small case series of 16 patients, the authors described that patients switched from type 1 to type 2 after 1 week of non-invasive support. Authors proposed that facing high respiratory drive, P-SILI is responsible to progression from type 1 to type 2 COVID-19 phenotypes.

Our initial experimental data suggest that one mechanism of the clinical observation of Gattinoni et al. may be due to regional lung volumetric deformation and pathological mechanotransduction induced by high strain-spontaneous breathing. As the lung does not discriminate the origin of the force that produce volumetric deformation, whether that can be generated by mechanical ventilation (VILI) or the respiratory muscles. Under this point of view, this last mechanism can be more precisely describe as “*Effort-induced lung injury*”, instead of P-SILI.

As some authors have pointed out, the type 1 and type 2 phenotypes are an oversimplification of ARF due to SARS-CoV2, as it is not possible to attribute to a single mechanism the complexity of COVID-19. Thus, respiratory support, non-invasive and invasive, cannot be decided on a single parameter to prevent potential complications and decrease morbidity and mortality.

Pathophysiology of COVID-19 respiratory failure [55] explains why patients with COVID-19 usually present with moderate to severe hypoxemia, so it seems appropriate to use standard oxygen therapy, HFNC and NIV as initial respiratory support. Due to the discordance of hypoxemia and respiratory distress, it is important to have in mind that previous studies that showed that stratified by severity hypoxemia high Vt (greater than 9.5 mL/kg [56] or 9 mL/kg [57]) predicts failure of NIV support. NIV failure has been associated to mortality [5], where high global strain may have a role on progression in lung injury. Interestingly, a study showed that the use of the helmet as an interface for NIV was associated with a better outcome than the traditional interface. Whether the possibility to deliver higher PEEP could be part of the explanation is not known [7]. High PEEP could reduce the respiratory drive, the negative pressure swings and global/regional strain due to caudal displacement and shortening of the diaphragm muscle. In this way, Sartini et al. recently described the effects of NIV and prone position cycles in patients with COVID-19 respiratory failure [58]. They found a significant decrease in respiratory rate and an improvement of oxygenation parameters.

It is impossible to asses isolated respiratory function in COVID-19 respiratory failure as well as other causes ARDS patients. A clear example in the study of Carteaux et al. where immunosuppression and severity also where associated to NIV failure [56]. As explained before, hypoxemia is infrequently the primary cause of respiratory distress, so it is important to consider other factors as well as non-respiratory organ disfunctions, like acute kidney injury, myocardial, and severe endothelial dysfunction; all of which are common in SARS-CoV2 [59–62]. The correct assessment of these factors gives a unique opportunity to non-respiratory treatments for COVID-19.

HFNC has shown remarkable results as primary respiratory support in de novo ARF [63], improving oxygenation and decreasing escalation of care and intubation rate when compared to standard oxygen therapy [63–65]. The benefit may result from the decrease of the anatomic dead space, reducing the ventilatory demand and work of breathing (WOB) [66]. In COVID-19, HFNC has been shown to be safe, well tolerated and it has a synergistic effect when combined with other treatments like prone position [67–71].

Prone position has been extensively studied in patients with ARDS and invasive MV, showing an improvement of oxygenation due to many mechanisms, like improving FRC, ventilation/perfusion heterogeneity, diaphragm motion in dorsal regions, increasing regional ventilation in dependent lung regions, among others [72, 73]. Some principles of prone position can be applied to awake extubated patients, but physiology is still not known in depth. It has been demonstrated as a safe intervention, and currently, it is widely used in emergency room, general wards as well as ICU settings [58, 68–71].

The physiological concepts of HFNC, NIV, and prone position can also be applied to patients with hypoxemic ARF secondary to SARS-CoV2 infection. Comorbidities are highly relevant in the selection of the selected non-invasive support strategy (i.e., morbid obesity, COPD, chronic heart failure). Awake prone position could attenuate P-SILI by reducing distending pressures, and negative swings of intrathoracic pressure, and more importantly, an increase in FRC. Theoretically, these mechanisms can improve alveolar interdependence phenomena by decreasing global strain and heterogeneity.

## A pragmatic approach to ARF due to SARS-CoV2

Some authors have highlighted the importance of a physiologic approach to SARS-CoV2 ARF [74–78]. We strongly recommend a conservative approach to respiratory failure due to SARS-CoV2. Hypoxemia alone (as well as all derived parameters, like P/F ratio) should not precipitate intubation, and PaO_2_ as low as 50 to 60 mmHg can be tolerated when there is no evidence of low end-organ perfusion or signs of dysoxia. All obvious indications of invasive MV, like hemodynamic instability, alteration of consciousness, should be carefully assessed over time. In our experience, in most of these patients, intubation can be prevented using timely non-invasive support, and treating identified or suspected complications early. A special consideration is to prevent fluid overload in these patients, although most of the time initial presentation is some degree of dehydration when acute kidney injury is not present. Off note is that CT-scans do not change our usual management. When patients develop an increase of WOB (respiratory rate greater than 35 breaths per minute, increase of respiratory muscles work, severe dyspnea and shortness of breath), a promptly evaluation of secondary causes is assessed and treated [78–81], including end-organ failure, early suspicion of bacterial superinfection, and thromboembolic events. This is in line with the concept of *multidimensional dyspnea* assessment coined by Banzett et al. [79, 81].

We acknowledge that there is no clear threshold to decide invasive MV, even considering hypoxemia and increased WOB. A special limitation is that there is no standardized measurement of respiratory distress in COVID-19 respiratory failure. Given these facts, there are many aspects of P-SILI still in debate and our current understanding is very limited on the role of P-SILI in progression of lung disease [78]. The tipping point might be the tolerance of the patient to non-invasive measures and the response to treatment, although it is also a subjective decision. MV is a lifesaving intervention in many situations, but it carries a high risk of complications. P-SILI during MV can occur in situations where high respiratory drive cannot be controlled. A short course of deep sedation and neuromuscular blocker (NMB), with daily assessment of discontinuation (NMB-holiday) is recommended [82, 83]. Weaning in ARF-COVID-19 also needs a special consideration. We have observed many situations where increase WOB due to high ⩒E demand, unrelated to the course of COVID-19 pneumonia, can prompt weaning failure, prolonging MV duration, or ultimately extubation failure. Usual situations include severe fever due to systemic unresolved inflammation, delirium, superinfection, drug withdrawal, and acidosis. Clinicians need to prevent them before weaning and extubation (i.e., early initiation of anti-psychotics, early discontinuation of benzodiazepines infusions, temperature control, etc.). All of these aspects are used to grasp our gestalt of ARF due to SARS-CoV2. It includes a multisystemic evaluation (not only respiratory system) to decide appropriate respiratory support, invasive or non-invasive, and correction of other factors that increased ⩒E demand and WOB [79]. After intubation, usual care to prevent complications are instituted [75, 77], and conditions for success weaning and extubation are assessed daily, to prevent an excessive duration of MV and morbidity and mortality associated [77, 82, 84].

## Conclusion

To our understanding, P-SILI might be one of the many factors that can explain progression of lung disease in COVID-19. We showed preliminary experimental data that regional lung strain and heterogeneity can be identified in acutely injured lungs under unassisted spontaneous breathing. They are capable to induce progression of lung collapse, inflammation, and progressive alterations of lung mechanics. Early use of controlled low Vt MV may prevent this progression. Given the current pandemic due to SARS-CoV2 and shortage of medical resources, like mechanical ventilators, the physicians must balance the interventions on respiratory support based on the pathophysiology of ARF, but also they must take into account that progression of disease severity might be a consequence of inadequate respiratory support in subjects with high work of breathing, even when it is not primary driven by respiratory failure.

## Data Availability

Not applicable
